# Preparing for pregnancy while living with HIV: An exploratory qualitative study of Women's preconception information needs and priorities

**DOI:** 10.1111/hiv.70304

**Published:** 2026-09-08

**Authors:** Catherine Stewart, A. Namiba, R. Mbewe, L. Kwardem, E. Ndungu, S. Tariq

**Affiliations:** ^1^ Institute for Women's Health University College London London UK; ^2^ 4M Network of Mentor Mothers London UK; ^3^ Institute for Global Health University College London London UK

**Keywords:** HIV, preconception, pregnancy

## Abstract

**Introduction:**

Preconception health is a key determinant of birthing people and infant outcomes. However, for people living with HIV, preconception care has predominantly focused on preventing vertical transmission, often neglecting broader needs. We conducted an exploratory qualitative descriptive study with women living with HIV to inform the development of a preconception health resource.

**Methods:**

In collaboration with HIV peer support organization 4M Network, we conducted five focus group discussions (FGDs) via Zoom in June–July 2024. Participants were eligible if they were living with HIV, aged ≥18 years, UK residents, and had either recently been pregnant (within 5 years) or were a 4M Mentor Mother or 4M Co‐Director. FGDs were recorded, transcribed and analysed thematically.

**Results:**

Sixteen cisgender women participated in five FGDs; aged 26–57, all Black/Mixed ethnicity, most non‐UK born, and diagnosed with HIV for 1–33 years. We identified five themes: healthy pregnancy meaning; pregnancy preparation; managing during pregnancy and postpartum; preconception discussions; and information sources. Participants across all groups (recently pregnant women, 4M Mentor Mothers and 4M Co‐Directors) identified a need for information on HIV management, physical and mental health, lifestyle, the pregnancy journey, and infant feeding. Most recently pregnant women had not discussed preconception health with their HIV clinicians, despite describing them as a main source of information and support. The findings emphasize the importance of holistic and tailored resources for women living with HIV.

**Conclusion:**

This study describes the diverse needs of women living with HIV as they prepare for pregnancy, highlighting how these needs extend beyond the prevention of vertical transmission of HIV. Our findings informed the development of a freely available preconception health resource for people living with HIV.

## INTRODUCTION

In 2022–2023 there were 534 pregnancies among people living with HIV in the United Kingdom (UK) [1]. Routine opt‐out antenatal HIV testing, appropriate management of delivery and infant feeding, and provision of antiretroviral therapy (ART) have contributed to substantial reductions in rates of vertical transmission of HIV to less than 0.4% in the UK [1].

Preconception health is a key determinant of maternal/birthing parent, pregnancy and neonatal outcomes [2, 3]. Preconception care refers to a range of strategic interventions delivered *before* conception to optimize health, reduce modifiable risk factors and improve the health and well‐being of birthing parents and children [4]. Good preconception care should be holistic, addressing a wide range of factors including management of long‐term conditions, nutrition and supplementation, lifestyle interventions and support for mental and social wellbeing [4, 5, 6].

People living with HIV have specific preconception health needs due to the lifelong management of a potentially stigmatizing condition and the higher prevalence of comorbidities, including hepatitis B/C, liver disease, diabetes, mental health conditions and cardiovascular, renal and bone disease [7, 8, 9]. People living with HIV also experience disproportionate socioeconomic challenges, for example, poverty, gender‐based violence and immigration‐related stressors [10]. Although rates of vertical transmission of HIV are low, rates of adverse pregnancy outcomes (e.g., preterm birth and low birth weight) are slightly higher than in the general population [1].

Despite additional clinical and social complexities, most published studies exploring preconception health in women living with HIV concentrate on ART, minimizing horizontal transmission during conception and preventing vertical transmission. Until 2025, UK guidelines on managing pregnancy and HIV mentioned preconception care only in relation to ART optimization [11]. While preventing vertical transmission remains essential, this narrow focus risks overlooking opportunities to support broader health and wellbeing among prospective parents living with HIV.

We aimed to explore and describe the information and support needs regarding preconception health and wellbeing among women living with HIV in the UK to inform the development of a tailored health resource.

## MATERIALS AND METHODS

This exploratory qualitative study comprised five online focus group discussions (FGDs) with cisgender women living with HIV in the UK in June–July 2024. The study was a collaboration with 4M Network (4M), a peer support organization that provides psychosocial support to peers in their pregnancy journey and beyond (https://4mmm.org/).

### Recruitment

We recruited participants via 4M's wide network of women living with HIV in the UK. Study information was shared through 4M's WhatsApp groups and social media. Interested individuals contacted CS to discuss involvement and were sent a participant information sheet.

Participants were eligible if they were: assigned female at birth, living with HIV, aged ≥18 years, living in the UK, able to consent, and if they had either been recently pregnant (within 5 years) *or* were a 4M Mentor Mother *or* a 4M Co‐Director. People who could not speak English were not eligible to participate due to the practical difficulties of using interpreters in FGDs.

Three FGDs were conducted with recently pregnant women (pregnant within 5 years), one with 4M Mentor Mothers, and one with 4M Co‐Directors. Mentor Mothers are women living with HIV who have previously experienced pregnancy and have received training from 4M to provide peer support, primarily on a voluntary basis. This training covers some aspects of pregnancy and HIV but focuses mainly on developing skills in providing peer support, personal development and achieving and maintaining good quality of life while living with HIV. 4M Co‐Directors, all women living with HIV, have extensive experience supporting women through pregnancy as well as close involvement with research, policy and advocacy.

The final sample size was guided by the concept of information power [12] and therefore data saturation was not sought or assessed. The sample was judged to be sufficiently information‐rich to address the focused study objective of understanding preconception health needs to inform the development of an information resource. Participants had direct lived experience relevant to the research question and contributed detailed accounts of preconception health needs. Pragmatic considerations of the project were also taken into account.

### Study procedures

All FGDs were conducted online via Zoom (participant preference) and facilitated by CS and AN/RM (4M staff, both black women living with HIV). FGDs lasted 60–90 mins and were digitally recorded. We collected anonymous demographic data including age, ethnicity, country of birth, years living in the UK, years since HIV diagnosis, relationship status and number of pregnancies.

We developed topic guides with 4M to ensure questions were appropriate and asked in a sensitive and non‐harmful way (Data [Link], [Link], [Link]). Our approach was informed by a strengths‐based approach, whereby ‘skills, knowledge and collective resources among marginalized communities’ are explicitly recognized and sought [13]. Minor adaptations were made to topic guides for each group to ensure relevance.

### Analysis

CS transcribed FGDs verbatim and analysed data thematically. An initial deductive coding framework was developed based on the topic guides. As analysis progressed, additional inductive codes were generated to capture unanticipated themes. The coding framework was discussed with all authors prior to being applied across transcripts. Data were managed using NVivo 14 [14].

Data from recently pregnant women, mentor mothers and 4M co‐directors were analysed separately and compared. We identified similar themes across all three groups and therefore synthesized findings while highlighting group‐specific differences where relevant.

### Positionality

The research team brought a range of lived, professional and clinical perspectives to the study. All authors are cisgender women, and most are from racially minoritized backgrounds. Several authors (AN, RM, EN and LK) are women living with HIV and have extensive experience of peer support, advocacy and reproductive health work within the HIV community. AN and RM also have lived experience of pregnancy while living with HIV. CS is a researcher with expertise in preconception health and care but is not living with HIV and has not experienced pregnancy. ST is a consultant HIV physician and public health academic who is not living with HIV but has personal experience of complex pregnancy and adverse pregnancy outcomes.

Our diverse backgrounds shaped both data collection and interpretation. CS facilitated all FGDs; as a white researcher without lived experience of HIV or pregnancy, she was mindful of how differences in identity and experience could influence participant engagement and group discussions. To support rapport, trust and openness, each FGD was co‐facilitated by a 4M Co‐Director with lived experience of HIV and pregnancy. This was particularly important when discussing potentially sensitive topics related to reproductive decision‐making, preconception health and experiences of HIV care.

## RESULTS

We recruited 16 participants: three 4M Co‐Directors, three 4M Mentor Mothers and 10 recently pregnant women. All participants were cisgender women living with HIV aged between 26 and 57 years. All identified as being of Black, Black British, Caribbean, African or mixed ethnicity and most (*n* = 12) had been born outside of the UK. Of those born abroad, duration of residence in the UK was between two and 35 years (median 16 years). Time since HIV diagnosis ranged from one to 33 years (median 6 years). Half of our participants were not currently in a relationship (*n* = 8) and all reported previous pregnancies (ranging from one to four). Age was the only main difference between the groups. Women who had recently been pregnant were younger (mean age 28.8 years) than mentor mothers (mean age 39.6 years) and co‐directors (mean age 53.0 years).

We identified five broad themes across the FGDs discussions: (1) the meaning of a healthy pregnancy; (2) pregnancy preparation; (3) managing during pregnancy and postpartum; (4) preconception discussions; and (5) information sources (Table 1).

**TABLE 1:** Matrix of findings by participant group.

	Women living with HIV	Mentor Mothers	4M Co‐Directors
Healthy pregnancy	Healthy baby Healthy mother (physical & mental health) Being informed & well supported	Healthy baby Healthy mother (physical & mental health) Being informed & well supported Addressing socioeconomic challenges	Healthy baby Healthy mother (physical & mental health) Being informed & well supported Addressing socioeconomic challenges
Preparing for pregnancy	Optimizing ART for pregnancy Optimizing ART adherence Optimizing physical and mental health Optimizing lifestyle – diet, vitamins, folic acid, exercise, stopping smoking drinking and recreational drugs	Optimizing ART for pregnancy Optimizing ART adherence Optimizing physical and mental health Optimizing lifestyle – stopping smoking, diet	Optimizing ART for pregnancy Optimizing ART adherence Optimizing physical and mental health Optimizing lifestyle – folic acid, diet Accessing appropriate financial support Accessing peer support
Managing during pregnancy and postpartum	Preparing a birth plan Being prepared for extra appointments Considering infant feeding decisions	Preparing a birth plan Considering infant feeding decisions	Preparing a birth plan Being prepared for extra appointments Considering infant feeding decisions
Preconception discussions	Discussions occur with HCP if initiated by woman Discussions with friends/family about pregnancy in general	No discussions about preconception in role as MM Discussions with friends/family about pregnancy in general	Discussions not common Discussions occur with HCP if initiated by woman
Sources of information about pregnancy and HIV	HIV clinician – facilitated by long term trusted relationships. Although some would not feel comfortable speaking to HIV clinician about pregnancy. Internet – Google and social media with trusted sources e.g. HIV organizations relied on more. Although some don't trust online sources.	N/A	N/A
Things to include in information resource	Acknowledge that every pregnancy is different What to expect on the journey e.g. extra appointments, ART for baby after birth Include infant feeding including reasons for why not breast/chest feeding and availability of free formula Highlight role of antenatal & support groups Signpost to resources e.g. support services Questions to ask at your appointment	Acknowledge that every pregnancy is different What to expect on the journey e.g. extra appointments, MDT Include infant feeding including reasons for why not breast/chest feeding and availability of free formula Highlight role of peer support/ support groups Signpost to resources e.g. doula service	Acknowledge that every pregnancy is different What to expect on the journey e.g. extra appointments Include infant feeding including reasons for why not breast/chest feeding and availability of free formula Highlight role of peer support/ support groups Signpost to resources e.g. doula services
Format of information resource	Multiple formats (online and hardcopy) Be able to be used in face‐to‐face consultations/discussions	Multiple formats (online and hardcopy) Be able to be used in face‐to‐face consultations/discussions Available in different languages	Multiple formats (online and hardcopy) Be able to be used in face‐to‐face consultations/discussions/mentor mother training

Abbreviations: ART, antiretroviral therapy; MDT, multidisciplinary team.

### The meaning of a healthy pregnancy

For the majority of participants across all groups, a healthy pregnancy was determined by the overall health of their baby, including prevention of vertical transmission of HIV. This was particularly salient within FGDs with recently pregnant women.

Well, basically for me I would say a healthy pregnancy is…me taking my pills [HIV medication] when I'm supposed to take them and watching my medication and my diet so that I don't in any way affect my baby. (FGD 2, recently pregnant woman)

However, participants in all groups also highlighted the importance of maternal health and how this was crucial for a healthy pregnancy. Maternal health was seen to encompass both physical and mental health, with mental health discussed in all FGDs, especially those with recently pregnant women.

Feeling like mentally well and then physically well as well… making sure that the HIV side of things was taken care of, and then the mental health side as well. (FGD 2, recently pregnant woman)

Participants in all groups viewed access to appropriate, tailored information and support before and during pregnancy as of central importance, with the need to be ‘well‐informed’ discussed in all FGDs:

A healthy pregnancy to me would be a pregnancy where you are…informed. (FGD 4, 4M Co‐Director)

### Pregnancy preparation

ART adherence was viewed by all groups as a key part of preparing for pregnancy when living with HIV. This theme was discussed most extensively in FGDs with recently pregnant women. Preventing vertical transmission was the prime motivation for adherence; however, participants in all groups also stressed the need to safeguard a woman's own health:

The most important preparation is taking your medication regularly. That's really what I think that, that would be the most important preparation to make you safe and baby safe as well. (FGD 1, recently pregnant woman)

The potential need to switch ART prior to conception, to ensure they were on regimens that were deemed safe and efficacious in pregnancy, was also discussed by participants in all groups.

I had to switch medications both times…because the medication wasn't, like, appropriate for pregnancy. So, knowing that I had to do that was quite helpful rather than maybe getting pregnant and having to switch…because it then gave me time to…get used to the medication. (FGD 2, recently pregnant woman)

Advice on lifestyle modification was raised by some participants in all groups, with specific reference to physical activity, diet, folic acid and giving up smoking, drinking and drug use.

Taking like folic acid, you know, just eating well, you know your diet. Just managing your body in a way, looking after your body so that it is ready to hold that baby. (FGD 5, 4M Mentor Mother)

Although the importance of improving health and wellbeing before pregnancy was highlighted in all FGDs by all groups of participants, detailed discussion of specific behaviours was relatively limited. The most detailed accounts of lifestyle modification came from recently pregnant women (Table 1).

Mental health optimization prior to pregnancy was a prominent and recurring theme in all five FGDs, across all participant groups. Mental health was consistently viewed as of equal importance to physical health and HIV management. Participants in all groups discussed how HIV could pose challenges to mental health because of stigma (including concerns about others knowing their HIV status) and additional decision‐making related to living with HIV.

Mentally…it's yeah, a very daunting thing in lots of cases and being pregnant with HIV wasn't really easy, the stigma and discrimination. (FGD3, recently pregnant woman)

For many participants in all groups, appropriate support before and during pregnancy was crucial to safeguarding emotional wellbeing and navigating the demands of pregnancy and parenthood.

Getting to handle your mental health as well is very, very important when preparing for pregnancy… getting a support group, having someone to speak to matters as well. (FGD 1, recently pregnant woman)

4M Co‐Directors highlighted the impact of broader social and structural factors when preparing for pregnancy. They emphasized the need for stability and security, including financial and housing security, freedom from domestic abuse and the resolution of immigration‐related concerns.

Being safe without…having to deal with stresses…so having somewhere to live…so that I can just focus on the pregnancy itself and not worry about … a violent partner…food poverty…financial poverty or immigration issues. (FGD4, 4M Co‐Director)

A small number of recently pregnant women mentioned these structural factors (in particular, financial and housing security and having a supportive partner), although this was a much less prominent topic of discussion.

### Managing during pregnancy and postpartum

Participants across the different groups did not see preconception as a distinct period; rather, it was part of a continuum of parenting that stretched from preconception to parenting:

Preparing for pregnancy for me, you know being able to, you know, take care of your child, being able to become a mother…it's just a preparation for motherhood. (FGD 3, recently pregnant woman)

Participants in all FGDs saw value in highlighting the importance of timely access to antenatal care and how their antenatal care may differ because of HIV:

Things like…what [antenatal care] would look like, because…you have extra appointments…your paediatrician is involved…so [you need to be prepared] so you're not like panicked when those conversations are being had once you are pregnant. (FGD 2, woman living with HIV)

Discussion of birth planning, including decisions around mode of delivery, emerged in all FGDs across all participant groups, and was considered an important aspect of pregnancy preparation.

But I think it's also really important to have…a birth plan in place and particularly for us, living with HIV. (FGD4, 4M Co‐Director)

Another central concern was infant feeding, and the decision whether to breast/chest feed or not.

You want to make sure [you have] like those discussions in terms of breastfeeding, yes you can, there is the option…but it's always a big discussion. (FGD1, recently pregnant woman)

While these topics are not directly related to preconception health, participants from all groups emphasized the value of being prepared for decisions that may need to be made during pregnancy, at an early stage.

### Preconception discussions

Participants in all FGDs described a lack of information and knowledge about how best to prepare for pregnancy:

Like that's one thing which I think is very, very key and isn't necessarily always taught. Because a lot of the time, it's like ok cool, it happens and then you've got to book a midwife and then you go to the hospital … but they don't necessarily give you the breakdown of all the information that you need beforehand. (FGD 1, recently pregnant woman)

Most recently pregnant women reported broad discussions about pregnancy with family and friends prior to conception, but not specific discussions about preparing:

I had conversations with my husband and my family before getting pregnant…about the idea of getting pregnant, the joy of being a mother. (FGD 2, recently pregnant woman)

Most recently, pregnant women described positive and supportive relationships with their HIV clinicians. Despite these trusted relationships, few reported discussing pregnancy with their HIV clinician (or another HCP) before conception. Where they occurred, these conversations were invariably initiated by the individual rather than the clinician and focused predominantly on ART and prevention of vertical transmission

When I ask the doctor, I was like … ‘will my baby be contracted with HIV? [Am] I going to deliver a safe baby, a healthy baby?’ (FGD 3, recently pregnant woman)

Only one participant (a recently pregnant woman) recalled discussing lifestyle changes and folic acid with their HIV clinician prior to conceiving.

I wanted to ask which vitamins or supplements I should start with before…my pregnancy. Then I also wanted to ask if I can change my lifestyle, maybe make any changes to my current medication. (FGD 3, recently pregnant woman)

4M Co‐Directors reiterated that discussions about pregnancy planning were often woman‐led rather than routinely initiated by healthcare professionals:

I think…for some it is discussed…it also differs from clinic to clinic in terms of what the clinicians…will discuss with you, but [they] will only do that if you express a wish that you're planning to get pregnant. (FGD 4, 4M Co‐Director)

### Information sources

Recently pregnant women differed in their preferred sources of information about pregnancy and preconception health. While some described online resources as their primary source of information, others identified their HIV clinician as their main source of support and advice. Women who reported strong, longstanding relationships with their HIV clinician were particularly likely to seek information from them, viewing them as a highly trusted and reliable source of pregnancy‐related information.

I had a really good relationship with my doctor, cause I'd known her for so long…we established that kind of relationship where we could talk about anything. (FGD1, recently pregnant woman)

These women also often expressed caution about online sources, citing concerns about accuracy and credibility.

I haven't really used online resources…I feel like especially when you use social media and like online, there's a lot of stuff which makes it a lot more scary. (FGD 1, recently pregnant woman)

However, other recently pregnant women (especially those without long‐term relationships with a clinician) reported relying heavily on online sources, including Google, Instagram, Facebook and TikTok. Accounts belonging to doctors and/or professional organizations were preferred. Convenience and privacy were highlighted as advantages of accessing information online, allowing them to seek information at a time and place that suited them and to explore topics that they may have felt uncomfortable raising with healthcare professionals.

You know, with our condition, it's not easy to walk into a particular place with HIV…so I just prefer being on my own in my house…just pick up your phone, search on Google or any other platform and you will have answers to what you're looking for. (FGD 2, recently pregnant woman)

There was broad agreement across all groups that tailored information on preparing for pregnancy was needed for people living with HIV. The vast majority of participants (in all groups) believed that information would need to be available in multiple formats. An online resource was seen as easy to access and share and could include links to additional information. However, a printed version that could be used in clinics and support services may facilitate face‐to‐face discussions. 4M Co‐Directors emphasized the importance of using clear, simple language and a visually engaging format, while Mentor Mothers suggested the resource should be translated into multiple languages.

## DISCUSSION

This exploratory descriptive study aimed to identify the preconception health information needs of women living with HIV to inform the development of a tailored information resource. To our knowledge, it is the first in the UK to explore this neglected area.

The findings highlight that for women living with HIV, a healthy pregnancy extends beyond having a baby born without HIV, although that remains the priority. Being well‐informed and supported to optimize health and wellbeing throughout the journey to and through parenthood was viewed by all groups as crucial for a positive experience. Our participants conceptualized preconception health holistically, highlighting ART (including the potential need to switch), physical and mental health, lifestyle modification, antenatal care, infant feeding, (peer‐) support services and what to expect during the pregnancy journey as areas to be discussed prior to getting pregnant. Of note, participants across all groups did not make a clear distinction between preconception, pregnancy and postpartum health, highlighting the additional appointments and decisions that accompany living with HIV as key things to be made aware of prior to getting pregnant.

A key finding is the gap between women's information needs and the information routinely provided. Despite established and trusted relationships with HIV clinicians, women who had recently been pregnant reported that discussions about preparing for pregnancy were uncommon and, when they occurred, were invariably initiated by women themselves and focused mainly on ART management and prevention of vertical transmission. However, our findings demonstrate that, while vertical transmission remains an important concern, women living with HIV have much broader informational and support needs that are currently unmet.

Our study has identified and responded to the need for an HIV‐specific resource on preconception health. Working in partnership with 4M Network and drawing directly upon our study findings, we developed the freely available resource ‘Preparing for Pregnancy While Living with HIV’ [15], which provides practical, evidence‐based information tailored to women living with HIV. Primarily intended as a patient‐facing resource, it can also be used to facilitate discussions with professionals. This resource is available in the Data S4, on 4M Network's website (https://4mmm.org/resources/preparing-for-pregnancy-while-living-with-hiv/) and is cited in the 2025 British HIV Association's guidelines on management of pregnancy and HIV [16].

This study has some limitations. As an exploratory project designed to inform resource development, the analysis was intentionally descriptive and did not seek to provide an in‐depth exploration of women's experiences. The study collected only limited sociodemographic information. Additional contextual information, such as geographical location, co‐existing health conditions and reproductive intentions, may have aided interpretation. Collaboration with 4M Network was a major strength, although recruitment through a single community organization may have limited the diversity of perspectives captured. Non‐English speakers were excluded due to the practical difficulties of using translators in FGDs. Furthermore, the sample included women who had recently experienced pregnancy, as well as 4M Mentor Mothers and 4M Co‐Directors. Although all participants were women living with HIV and brought relevant lived experience, the Mentor Mothers may have had greater engagement with healthcare services, and their peer‐support training may have shaped their views on preconception health. Similarly, the Co‐Directors' involvement in research, policy and advocacy may have provided them with additional knowledge and influenced their perspectives. Consequently, findings may not fully reflect the experiences and information needs of all women living with HIV across the UK. Focus groups may be subject to group dynamics and social desirability effects; however, this method is well established in the study of sensitive topics [17]. Finally, the analysis of the focus groups was led by a single researcher, increasing the potential for subjective interpretation. However, codes were discussed with the wider research team prior to being applied to all transcripts.

A key strength of this work is its focus on preconception information needs among women living with HIV, a notable gap in the existing literature. The study was fundamentally co‐produced; women living with HIV contributed to study design, interpretation of findings, and development of outputs. This helped ensure the research remained relevant to the community it was intended to serve. A further strength is the direct translation of findings into practice through the design of a tailored preconception health resource for women living with HIV, the first of its kind in the UK.

## CONCLUSION

This exploratory qualitative study highlights the wide‐ranging information needs of women living with HIV as they prepare for pregnancy, extending beyond vertical transmission prevention. Participants across all groups identified a need for information on HIV management, physical and mental health, lifestyle, social support, antenatal care and infant feeding. The findings emphasize the importance of holistic, tailored and accessible resources that reflect pregnancy planning as part of a broader continuum, rather than a distinct phase. In response, we developed the freely available resource ‘Preparing for Pregnancy While Living with HIV’ with 4M Network, which is now cited in national guidelines and used by community organizations.

## AUTHOR CONTRIBUTIONS

**CS:** Conceptualization, Methodology, Investigation, Project administration, Data curation, Formal analysis, Validation, Visualization, Writing – original draft, Writing – review & editing, **ST:** Conceptualization, Methodology, Validation, Supervision, Writing – review & editing, **AN:** Methodology, Investigation, Validation, Writing – review & editing, **RM:** Methodology, Investigation, Validation, Writing – review & editing, **LK:** Methodology, Validation, Writing – review & editing, **EN:** Investigation, Writing – review & editing. All authors contributed to the development and final version of the informational resource.

## FUNDING INFORMATION

This work and CS time was funded by a Medical Research Council (MRC) PhD grant (MR/W006774/1).

## CONFLICT OF INTEREST STATEMENT

The authors declare no conflicts of interest.

## ETHICS STATEMENT

This study has ethical approval from UCL's Research Ethics Committee (REF 26901/001). All participants provided written informed consent and were given a £25 shopping voucher in recognition of their time and emotional labour.

## Supporting information


**Data S1.** Topic Guide for FGDs with Recently Pregnant Women.

**Data S2.** Topic guide for FGDs with Mentor Mothers.

**Data S3.** Topic guide for FGD with Co‐directors.

**Data S4.** Preparing for pregnancy while living with HIV.

## Data Availability

Research data are not shared.
