# Supplementary material for: Preparing for pregnancy while living with HIV: An exploratory qualitative study of Women's preconception information needs and priorities
Source: HIV Med. 2026 Sep 8;27(11):1823–31. doi: 10.1111/hiv.70304 (PMC13725711; doi:10.1111/hiv.70304)
Supplement: Supplementary file 1 [file HIV-27-1823-s002.pdf]

PREPARING FOR PREGNANCY
WHILE LIVING WITH HIV

THINGS TO DO BEFORE GETTING PREGNANT

- Discuss HIV & medication with doctor
- Discuss other health conditions with doctor
- Start taking folic acid
- Have a healthy lifestyle

THINGS TO THINK ABOUT BEFORE GETTING PREGNANT
(OR ONCE YOU ARE PREGNANT)

- Support
- Appointments
- Birth Plan
- Infant Feeding

GETTING PREGNANT

- Getting Pregnant

4M Network
My health, My choice, My child, My life
UCL

THINGS TO DO BEFORE GETTING PREGNANT
WHILE LIVING WITH HIV

HIV & Medication

People living with HIV can have safe pregnancies and healthy children (who do not have HIV themselves).

Before getting pregnant, it is important to speak to someone in your HIV healthcare team to discuss getting pregnant so they can make sure you are on the right HIV medication. Most HIV medication is safe to use during pregnancy. Some medication may need to be changed if there's not enough information about using them in pregnancy, or if they don't work as well in pregnancy.

It's important to **take your HIV medication regularly, as prescribed,** before you get pregnant, during pregnancy and afterwards. This is important for you and your baby's health. Being undetectable means that you'll be in the best health possible and that the chances of passing HIV on during or after pregnancy is very low.

If you are **not** currently taking HIV medication, **please speak to your HIV clinic** to discuss your options before getting pregnant.

Questions you might want to ask your clinic –

- How will HIV affect my pregnancy or the health of my baby?
- Do I need to change my HIV medication before getting pregnant?
- If I change medication am I likely to feel any different?
- What do I do if I experience any issues with my new medication?
- What support is there for me as I try to get (or once I become) pregnant?

More information about HIV medication and pregnancy:

- Planning a Pregnancy with HIV
- BHIVA Pregnancy Guidelines
- i-Base HIV & Pregnancy Guides

THINGS TO DO BEFORE GETTING PREGNANT
WHILE LIVING WITH HIV

Health conditions

Most people who have medical conditions (including HIV) before pregnancy have healthy pregnancies and babies. However, you may need some extra appointments and/or tests to help keep you and your baby safe.

If you have **any other medical conditions** it is important to **talk to your HIV clinic or GP before getting pregnant.** They will discuss how you can prepare for pregnancy and be the in the best possible health. Examples of other conditions include:

- Diabetes
- High blood pressure
- Asthma
- Heart conditions
- Epilepsy
- Thyroid disorders
- Mental health conditions

Some non-HIV medicines (both prescribed and over the counter) may not be safe in pregnancy. It's important to **discuss any medications you take with you doctor/pharmacist, but don't stop taking medication without advice.**

Questions to ask your HIV clinic, GP or pharmacist:

- Is there anything different I need to do to manage my medical condition before getting pregnant?
- Do I need to change my medication before or during pregnancy?
- Will my health condition change the way my pregnancy is managed?
- Will pregnancy make my medical condition worse?
- Do I need to take a higher dose of folic acid?

Visit Tommy's to find out more about how health conditions affect pregnancy.

THINGS TO DO BEFORE GETTING PREGNANT
WHILE LIVING WITH HIV

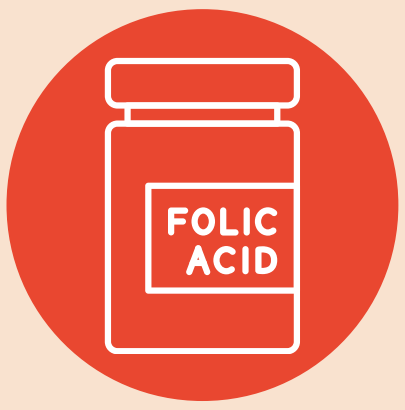

Folic Acid & Supplements

Folic acid is a vitamin that helps with the development of a baby's brain, skull and spinal cord. It is important to **take folic acid every day for at least three months before becoming pregnant and until you're 12 weeks pregnant.**

It is recommended to take **400mcg of folic acid daily**. However, if you have diabetes, are on some epilepsy medications, are living with obesity or have previously had a baby with a neural tube defect, you should **discuss a higher dose with your GP.**

Other supplements recommended before & during pregnancy:
10 mg Vitamin D (to help your baby's bones grow strong)
150 mcg Iodine (to help with the development of your baby's brain and nervous system)

You can get folic acid, vitamin D and iodine either as part of a pregnancy multivitamin (make sure the multivitamin **doesn't** contain Vitamin A) or individually (which is often cheaper) from most high street pharmacists or supermarkets.

If you qualify for the Healthy Start Scheme you may be able to access some vitamins for free once you are pregnant.

If you require a higher dose of folic acid, you can get this prescribed for free from your GP.

Learn more about folic acid.
Learn more about supplements and nutrients in pregnancy
Learn more about the Healthy Start Scheme.

THINGS TO DO BEFORE GETTING PREGNANT
WHILE LIVING WITH HIV

Lifestyle changes

Your health before pregnancy can affect your chances of getting pregnant as well as your pregnancy and the health of your baby.

Before getting pregnant, it is recommended to:

- **Stay active** (at least 150 mins of movement a week) e.g. walking, cycling, swimming or running.
- **Eat at least 5 portions of fruit and vegetables a day**
- **Achieve a healthy weight** (measure your waist and divide by your height – under 0.5 is in the healthy range)
- **Reduce caffeine** (to below 200mg a day, see 'caffeine calculator' link below)
- **Avoid alcohol**
- **Quit smoking/vaping**
- **Stop using recreational drugs**

Visit Tommy's to find out more about preparing for pregnancy and try their 'Planning for Pregnancy' tool to find out how to improve the health of your future pregnancy and baby. **If you need help changing your lifestyle then speak to your HIV clinic or GP.**

More information about staying healthy:

NHS Get Active
NHS Fitness Video
NHS Healthy Recipes
NHS 5 a day
Healthy Start Scheme
Caffeine Calculator
Alcohol Support
Kwit: An app to help people quit smoking
NHS Stop Smoking Services
NHS Drug Support

THINGS TO THINK ABOUT BEFORE GETTING PREGNANT
WHILE LIVING WITH HIV

Support

Throughout your pregnancy you will be cared for by a team of specialists. Who is in the team may vary by hospital but usually includes:

- an HIV doctor
- an obstetrician (who focuses on pregnancy and birth)
- a specialist midwife
- a paediatrician (who focuses on child health)

All of your healthcare team needs to be aware that you have HIV (so they can care for you and your baby properly). They should not tell anyone else about your HIV status without your permission. You may want to talk to your team so they know who knows about your HIV status (and who doesn't) so your wishes are respected and that information isn't shared without your permission.

Antenatal Classes

These classes can help you prepare for childbirth and provide information about parenting. The NHS provide free antenatal classes. Most start when you're around 30 weeks pregnant, however they can book up fast, so it's a good idea to look into this early. Some hospitals may provide antenatal classes specifically for women living with HIV so speak to your midwife.

- Find out more about NHS Antenatal Classes here
- Find your local Antenatal Classes here.

Doula

You can also choose to have the support of a Doula, a professional trained in supporting childbirth. This is usually not a free service. Doulas provide emotional, physical, and educational support to parents during pregnancy, labour and after birth. They help people have a safe, memorable, and empowering birthing experience. Some doulas may have specific expertise in HIV.

- Learn more about the services offered by a doula by watching 4M Webinar on: An introduction to and the benefits of a doula
- Contact 4M for recommendations for doulas with expertise in HIV.

THINGS TO THINK ABOUT BEFORE GETTING PREGNANT

WHILE LIVING WITH HIV

Support

Peer support
Peer support is where people with similar experiences connect and share experiences and knowledge, and provide practical assistance. **4M Network** can provide peer support throughout your pregnancy journey (and beyond). 4M trains women living with HIV to become Mentor Mothers. They are a great source of information, whether you have a specific question you want answered or if you just want to be connected with others who've had similar experiences.

Contact 4M Network.

You should be offered peer support and asked about your mental health by your healthcare team throughout pregnancy and after birth

Appointments

Throughout pregnancy you should be cared for by a team of specialists. In addition to standard scans and appointments offered to every pregnant person, **you will also be offered extra heath checks.**

Example of extra appointments you might have:

- HIV specialist consultations throughout to discuss health, medication, birth plan and any concerns
- Regular blood tests to monitor HIV viral load
- Post-birth check (within 24 hours of birth) to test the baby for HIV and give the baby HIV medication
- HIV testing of your baby at 6 weeks, 12 weeks, 18 months
- If you choose to breast/chestfeed, you'll also have monthly blood tests for you and your baby to check HIV viral load.

These are just examples of some of the extra appointments you may have during pregnancy, everyone's schedule will look slightly different.

THINGS TO THINK ABOUT BEFORE GETTING PREGNANT
WHILE LIVING WITH HIV

Birth Plan

It can be useful to think about your birth before getting pregnant – thinking about how you'd like to give birth (vaginal or C-section), who you want with you when giving birth (birth partner), pain relief, birthing equipment (such as birthing pool), positions for labour and skin-to-skin contact with your baby.

Plans for birth need to be flexible as things can change during pregnancy or during the birth. This can be disappointing but it is important to make sure you and your baby are safe.

Discussing how you'd like to give birth with your healthcare team will help you balance risks and benefits of different types of delivery.

You will be asked to give your baby HIV medication for up to 4 weeks after birth to reduce the chance of HIV being transmitted. If you have any concerns you can speak to your healthcare team.

Infant Feeding
(Breast/chestfeeding or formula feeding)

Currently, in the UK, it is recommended that people living with HIV bottle feed their baby using formula milk. This means there is zero chance of HIV being passed on through breastmilk. However, the guidelines also say that **anyone who wishes to breast/chestfeed should be supported to do so.**

There is a lot of information to help with this decision (see 'Useful Links' section). Your healthcare team can also give you lots of advice on how to breast/chestfeed safely.

If you choose NOT to breast/chestfeed you might be able to access free formula milk. Your midwife can give you information.

Choosing not to breast/chestfeed can prompt questions from family members or friends. If you choose not to breastfeed it may be helpful to have a 'reason' for why you're not breastfeeding. Your healthcare team can give you examples e.g. 'My milk hasn't come in', 'I have a low milk supply'. You may also be able to take a drug after birth to stop you producing milk.

GETTING PREGNANT
WHILE LIVING WITH HIV

Getting Pregnant

Before getting pregnant, it is important to talk to your HIV clinic about pregnancy, medication and your options.

Undetectable = Untransmittable (U=U) means that if you are taking medication and have a fully supressed viral load you have zero risk of passing the virus to your sexual partner. You can have sex wihout a condom to get pregnant without passing HIV on to your partner. This means you need to take your HIV medication everyday as prescribed so that you stay undetectable.

If you (or your partner) don't have an undetectable viral load, it is important that you speak to your HIV clinic before trying to get pregnant to discuss your options for conceiving safely.

The menstrual cycle is a monthly process – it is usually 28 days but it can vary. Ovulation is the release of an egg from your ovaries, it generally occurs in the second half of your cycle. Your most fertile period is around the time of ovulation, this is when you're most likely to conceive.

To help you figure out when you are ovulating, it can be useful to:

- Track your menstrual cycle: Rrecord the start and end dates of your period each month to understand your cycle's length and regularity
- Monitor symptoms throughout your cycle: pay attention to signs like breast tenderness or slight cramping, which can indicate ovulation
- Use an ovulation test kit: these kits can detect hormone changes, and your most fertile days. Ovulation test kits can be bought at most pharmacies

If you've been trying to get pregnant for 12 months without success, you should visit your GP to discuss your options.

Once you're pregnant, it's important to contact your HIV clinic as soon as possible. They will refer you to your local maternity service, where you will be looked after by your team of specialists.

USEFUL LINKS

Useful Links

Peer Support

- 4M Network

Planning a Pregnancy

- Planning a Pregnancy with HIV
- BHIVA Pregnancy Guidelines
- i-Base HIV & Pregnancy Guides
- Health Conditions and Pregnancy
- Folic Acid
- Supplements and nutrients in pregnancy
- Healthy Start Scheme
- NHS Get Active
- NHS Fitness Video
- NHS Healthy Recipes
- NHS 5 a day
- Caffeine Calculator
- Alcohol Support
- Kwit: An app to help people quit smoking
- NHS Stop Smoking Services
- NHS Drug Support

Getting Pregnant

- U=U Frequently Asked Questions
- How to get pregnant
- Ovulation calculator

Pregnancy

- NHS Birth Plan
- NHS Antenatal Classes
- Find your local Antenatal Classes
- 4M Webinar on: An introduction to and the benefits of a doula

Breast/Chestfeeding

- 4M's Breastfeeding Paper
- BHIVA Breastfeeding Leaflet

GETTING PREGNANT
WHILE LIVING WITH HIV

Research

This informational resource on preparing for pregnancy while living with HIV has been created from research conducted by researchers at UCL in collaboration with 4M Net.

Authors: Catherine Stewart, Estelle Ndungo, Longret Kwardem, Rebecca Mbewe, Angelina Namiba, Shema Tariq

Focus group discussions were conducted with women living with HIV, to understand more about the needs and concerns they have when thinking about preparing for pregnancy. The main topics discussed within those focus groups have been included in this resource.

We would like to thank everyone who took part in our focus groups and sharing their experiences and opinions.

The research received ethical approval (Ethics ID: 26901/001).

Funded by MRC (grant reference MRC/W006774/1).
