# Supplementary material for: Preparing for pregnancy while living with HIV: An exploratory qualitative study of Women's preconception information needs and priorities
Source: HIV Med. 2026 Sep 8;27(11):1823–31. doi: 10.1111/hiv.70304 (PMC13725711; doi:10.1111/hiv.70304)
Supplement: Supplementary file 2 [file HIV-27-1823-s001.docx]

**Exploring preconception health among women living with HIV in the UK**

**Topic Guide for FGDs with Mentor Mothers.**

*Note to facilitators: Instructions to facilitators are in standard print. Questions to read out are in* ***bold****. Prompts are also provided, to be read out if and when needed (for example, if participants do not understand a question, or to help encourage further discussion).*

**SET UP FOR THE FOCUS GROUP**

Step 1: Setting up for the Session

1. There should be two facilitators: 1 to lead discussion and 1 to provide support.
2. Ensure online white board is set up.

Step 2: Upon arrival of Focus Group Participants

1. Ensure each participant has had an information sheet, the opportunity to ask questions and has signed the consent form.

Step 3: Introductions (5 minutes)

1. Welcome, introduce self and facilitator, explaining they will be taking notes and recording the session.

**Introductions**

**“We will be recording this discussion using the Zoom recording function, but this will only be listened to by me and by members of the team and it’s just so that I can remember what you said. No-one else can access the recording and it will stored in the UCL Data Safe Haven, a secure portal that ensures only those with access can view the recording**”.

Reiterate the purpose of the discussion:

**“I’m very grateful to you for sparing time to talk to me today. The aim of the discussion today, is to understand more about what the support needs are of women living with HIV as they prepare for pregnancy, so we can develop an informational resource that can be used by 4M. There are no right or wrong opinions, I would like you to feel comfortable saying what you really think and how you really feel. Please don’t feel you have to answer anything you don’t want to and please let us know if wish to stop at any point. Finally, we would like to ask you not to share anything we discuss here today outside this room so people feel comfortable sharing their thoughts and experiences.”**

**Section 1: Experiences of preparing for pregnancy with HIV**

**Q1. Can we start by going round and introducing ourselves, and also telling us a bit about your role as a Mentor Mother?**

- Start with facilitators
- How long have you been a 4M Mentor Mother?

**Q2. What does a healthy pregnancy mean to you?**

**Q3. Can you share any thoughts you have about what ‘preparing for pregnancy’ means?**

- How do you think living with HIV impacts that?

**Section 2: Support and Information**

**Q4. In your experience supporting women living with HIV, is health before pregnancy something that is important to women?**

- How has HIV impacted their experience of thinking about/preparing for pregnancy (if it has)?
- Do you think HIV makes it easier/harder/the same?

**Q5. Where do you get the information you share with women from?**

- What are trusted sources of information?
- What kind of resources do you use?
- What sources do women seem to prefer?

**Section 3: Creating an informational resource for women living with HIV on preparing for pregnancy**

**“UCL and 4M are working together to develop an informational resource on preparing for pregnancy for women living with HIV. We’d really like you to help us make sure this informational resource is tailored to the needs of women living with HIV.”**

**Q6. What do you think about this idea?**

- Would it be useful?
- What format would you like this resource to be in?
- When and how do you think you would use this resource?
- Any examples of good resources you have used in the past?

Activity 1: Questions about preparing for pregnancy

Open online whiteboard

**Could you please take some time and please write on the whiteboard some of the most common things you get asked by women living with HIV who are thinking about or planning to get pregnant? Or any questions you have about preparing for pregnancy while living with HIV that you would like answers to, to better support women living with HIV?**

**Can we now come back as a group. Would you like to share what you wrote on the whiteboard?**

**Q7. We’re reaching the end of the group discussion now. Is there anything else you would like to add that we haven’t covered yet?**
