# Supplementary material for: Preparing for pregnancy while living with HIV: An exploratory qualitative study of Women's preconception information needs and priorities
Source: HIV Med. 2026 Sep 8;27(11):1823–31. doi: 10.1111/hiv.70304 (PMC13725711; doi:10.1111/hiv.70304)
Supplement: Supplementary file 3 [file HIV-27-1823-s003.docx]

**Exploring preconception health among women living with HIV in the UK**

**Topic Guide for FGD with Co-Directors.**

*Note to facilitators: Instructions to facilitators are in standard print. Questions to read out are in* ***bold****. Prompts are also provided, to be read out if and when needed (for example, if participants do not understand a question, or to help encourage further discussion).*

**SET UP FOR THE INTERVIEW**

Ensure the participant has had an information sheet, the opportunity to ask questions and has signed the consent form.

Introductions

**“We will be recording this discussion using the Zoom recording function, but this will only be listened to by me and by members of the team and it’s just so that I can remember what you said. No-one else can access the recording and it will stored in the UCL Data Safe Haven, a secure portal that ensures only those with access can view the recording”.**

Reiterate the purpose of the discussion:

**“I’m very grateful to you for sparing time to talk to me today. The aim of the discussion today, is to understand more about what the support needs are of women living with HIV as they prepare for pregnancy, so we can develop an informational resource that can be used by 4M. There are no right or wrong opinions, I would like you to feel comfortable saying what you really think and how you really feel. Please don’t feel you have to answer anything you don’t want to and please let us know if wish to stop at any point. Finally, we would like to ask you not to share anything we discuss here today outside this room so people feel comfortable sharing their thoughts and experiences.”**

Section 1: Experiences of preparing for pregnancy with HIV

**Q1. Can we start by introducing ourselves and telling me a little more about your role at 4M.**

**Q2. What does a healthy pregnancy mean to you?**

**Q3. Can you share any thoughts you have about what ‘preparing for pregnancy’ means?**

- Main ways to prepare for pregnancy
- How do you think living with HIV impacts that?

**Section 2: Support and Information**

**Q3. In your experience supporting women living with HIV, is preparing for pregnancy something that most women do? Or is important to women?**

- How does living with HIV impact the experience of preparing for pregnancy (if it has)?
- Do you think HIV makes it easier/harder/the same?
- Main barriers to preparing for pregnancy?
- Is HIV a barrier to preparing for pregnancy?

**Q4. Where do you get the information you share with women from? Or where do women get their information from?**

- What are trusted sources of information?
- What kind of resources do you use?
- What sources do women seem to prefer?
- Online resources?

**Section 3: Creating an informational resource for women living with HIV on preparing for pregnancy**

**UCL and 4M are working together to develop an informational resource on preparing for pregnancy for women living with HIV. We’d really like you to help us make sure this informational resource is tailored to the needs of women living with HIV.**

**Q5. When and how do you think this resource would be used?**

- What format would you like this resource to be in? e.g. leaflet, webinar?

**Q6. What are the main issues/concerns that women, living with HIV, have during the preconception period that could be addressed in this resource?**

**What are some of the most common things you get asked by women living with HIV who are thinking about or planning to get pregnant? Or any questions you have about preparing for pregnancy while living with HIV that you would like answers to, to better support women living with HIV?**

**Q7. We’re reaching the end of the FGD now. Is there anything else you would like to add that we haven’t covered yet?**
