# Supplementary material for: Preparing for pregnancy while living with HIV: An exploratory qualitative study of Women's preconception information needs and priorities
Source: HIV Med. 2026 Sep 8;27(11):1823–31. doi: 10.1111/hiv.70304 (PMC13725711; doi:10.1111/hiv.70304)
Supplement: Supplementary file 4 [file HIV-27-1823-s004.docx]

**Exploring preconception health among women living with HIV in the UK**

**Topic Guide for FGDs with Recently Pregnant Women.**

*Note to facilitators: Instructions to facilitators are in standard print. Questions to read out are in* ***bold****. Prompts are also provided, to be read out if and when needed (for example, if participants do not understand a question, or to help encourage further discussion).*

**SET UP FOR THE FOCUS GROUP**

Step 1: Setting up for the Session

1. There should be two facilitators: 1 to lead discussion and 1 to provide support.
2. Ensure online whiteboard is set up and break-out rooms are assigned.

Step 2: Upon arrival of Focus Group Participants

1. Ensure each participant has had an information sheet, the opportunity to ask questions and has signed the consent form.

Step 3: Introductions (5 minutes)

1. Welcome, introduce self and facilitator, explaining they will be taking notes.

Introductions

**“We will be recording this discussion using the Zoom recording function, but this will only be listened to by me and by members of the team and it’s just so that I can remember what you said. No-one else can access the recording and it will stored in the UCL Data Safe Haven, a secure portal that ensures only those with access can view the recording”.**

Reiterate the purpose of the discussion:

**“I’m very grateful to you for sparing time to talk to me today. The aim of the discussion today, is to understand more about what the support needs are of women living with HIV as they prepare for pregnancy, so we can develop an informational resource that can be used by 4M Mentor Mothers. There are no right or wrong opinions, I would like you to feel comfortable saying what you really think and how you really feel. Please don’t feel you have to answer anything you don’t want to and please let us know if wish to stop at any point. Finally, we would like to ask you not to share anything we discuss here today outside this room so people feel comfortable sharing their thoughts and experiences.”**

Section 1: Experiences of preparing for pregnancy with HIV

**Q1. Can we start by going round and introducing ourselves, and also telling us a bit about why you wanted to join this discussion group today?**

- Start with facilitators

**Q2. What does a healthy pregnancy mean to you?**

**Q3. Can you share any thoughts you have about what ‘preparing for pregnancy’ means?**

- What things would you want to have in place before getting pregnant (either for the first time or again)
- How do you think living with HIV impacts that?

**Q4. Can you share your own experiences of preparing for pregnancy, while living with HIV?**

- How has HIV impacted your experience of preparing for pregnancy (if it has)?
- Do you think HIV makes it easier/harder/the same?

Section 2: Support and Information

**Q5. Before getting pregnant, did you speak to someone about preparing for pregnancy?**

- A healthcare provider/friend/family member/Mentor Mother?
- What did you ask?
- How confident were you with the information provided?
- Why haven’t you spoken to a healthcare professional? What are the barriers?
- Did you initiate discussions or did they?
- Would you want a healthcare professional to ask about pregnancy intentions in regular checkups?

Activity 1: Support / information about preparing for pregnancy

Open online whiteboard with list of information sources on.

**Could you please, as a group, rank these sources of information from most used to least used.**

- Thinking about access, approachability, ease, trustworthiness and supportiveness.

1. HIV specialist doctor
2. GP
3. Mentor Mother
4. Online sources
5. Social media
6. Friends and family
7. Other (please specify)

**Can we now come back as a group. Would you like to share your ranking and discussions?**

Section 3: Creating an informational resource for women living with HIV on preparing for pregnancy

**“UCL and 4M are working together to develop an informational resource on preparing for pregnancy for women living with HIV, that 4M will be able to use to support their network. We’d really like you to help us make sure this informational resource is tailored to your needs.”**

**Q6. What do you think about this idea?**

- Would it be useful?

Activity 2: Questions about preparing for pregnancy

**Can you now take some time and please think about any questions you have about preparing for pregnancy while living with HIV that you would like answers to? Please write any questions either in the chat or just speak them.**

**Can we now come back as a group. Would you like to share a question you have?**

**Q7. When and how you would like this information to be shared with you?**

**Q8. Is there anything you would like to be included in this resource that we haven’t previously discussed?**

**Q9. We’re reaching the end of the group discussion now. Is there anything else you would like to add that we haven’t covered yet?**
